# Biologically Unrelated Living Donor for Kidney Transplantation Associated With Higher Acute Rejection Rate but With Similar Graft Survival of a Related Living Donor

**DOI:** 10.7759/cureus.30189

**Published:** 2022-10-11

**Authors:** Pedro Reis Pereira, Manuela Almeida, Bárbara Ribeiro, João Oliveira, Luisa Costa, Sofia Pedroso, La Salete Martins, Leonídio Dias, Jorge Malheiro

**Affiliations:** 1 Nephrology, Centro Hospitalar de Trás-os-Montes e Alto Douro, Vila Real, PRT; 2 Nephrology, Dialysis, and Transplantation, Unit for Multidisciplinary Research in Biomedicine – Instituto de Ciências Biomédicas Abel Salazar (ICBAS), Porto, PRT; 3 Integrative and Translational Research, ITR - Laboratory for Integrative and Translational Research in Population Health, Porto, PRT; 4 Nephrology, Centro Hospitalar Universitário Porto, Porto, PRT; 5 Nephrology, Hospital de Braga, Braga, PRT; 6 Nephrology, Centro Hospitalar Universitário do Porto, Porto, PRT; 7 Nephrology, Centro Hospitalar Tondela-Viseu, Viseu, PRT

**Keywords:** living-unrelated donor, immunosuppression, acute rejection, graft survival, kidney transplant, living-related donor

## Abstract

Introduction: Kidney transplantation (KT) from living donors has been shown to have multiple benefits compared to those from deceased donors. We sought to compare significant graft outcomes, namely acute rejection (AR), graft function, and survival between transplant recipients who received a kidney from living related donor (LRD) and living unrelated donor (LURD).

Methods: Our cohort comprised 198 donor and recipient pairs undergoing living-donor KT at our center over 10 years. The LRD recipients were compared with LURD recipients according to demographic and clinical characteristics, transplant variables (including immunosuppression), graft function, survival, and AR rate.

Results: The estimated glomerular filtration rate (eGFR) was similar in both groups over the follow-up time i.e., 60-65 mL/min (p>0.05 over 10 years). Censored graft survival was similar between LRD and LURD recipients (96.9% vs. 98.0% at five years and 87.8% vs. 79.4% at 10 years, respectively; p=0.837). The LURD recipients had a higher incidence of AR, although LURD recipient status was not an independent risk factor for AR. Multivariate analysis showed that human leukocyte antigen (HLA)-DR mismatch (MM) was an independent predictor of AR (hazard ratio (HR) 2.256, p<0.05). Both HLA-A and HLA-B MM did not affect the AR HR between the groups.

Conclusion: Graft function and censored graft survival rates were similar between LURD and LRD KT recipients in our study. The AR was higher in LURD recipients, although the LURD recipient status was not an independent risk factor for AR. The HLA-DR MM was an independent predictor of AR, while HLA-A and HLA-B MM did not affect AR HR between groups of patients.

## Introduction

Kidney transplantation (KT) has been shown to improve survival and long-term outcomes in patients with end-stage kidney failure [1]. Over the last decades, the number of patients on the KT waitlist has been steadily increasing [2]. In the setting of organ scarcity, living KT allows for an increase in the donor pool and reduces waiting time for KT [3].

Living donors can be classified as living related donors (LRD) or as living unrelated donors (LURD). An LRD is defined as being genetically related to the transplant recipient, such as parents, siblings, or children. A LURD, on the other hand, is not genetically related to the transplant recipient. However, they could be someone with whom the recipient has an emotional connection, such as a spouse or a friend, or an unacquainted person, such as an altruistic donor or a donor from a kidney paired exchange (KPE) program. With policies and legislative issues varying between countries, some countries do not allow LURD KT or KPE programs. In Portugal, legislation allowing genetically unrelated transplantation was passed in 2007, and is based on evidence showing that transplants from unrelated living donors too, have better outcomes compared with transplants from deceased donors [4].

Several studies have focused on comparing the outcomes between LRD and LURD transplant recipients. Most studies have shown similar graft survival between recipients of these two types of living donation [5-11], while some studies have shown a better survival of LRD recipients compared to LURD recipients [12,13]. A recent study with 14,370 patients reported similar patient and overall graft survival in LRD and LURD recipients, while a higher death-censored graft failure in LURD recipients was noticeable [14]. Moreover, some studies have reported higher rates of vascular rejection in LURD recipients [10,15], while others observed similar rates of acute rejection (AR) between both types of living KT [8]. Incidence of chronic allograph nephropathy has also been shown not to be different between LRD and LURD recipients, as well as rates of other post-transplant complications [15]. 

In this study we aimed to compare graft function and survival, as well as rates of AR in transplant recipients from LRD and LURD, evaluating the first 10 years of our center’s experience after the introduction of LURD KT in Portugal.

## Materials and methods

Study population

We retrospectively reviewed the clinical data of adult donor and recipient pairs undergoing living donor KT (LDKT) at our institution between January 2008 and December 2017 (n=210). After the exclusion of seven recipients who had been submitted to ABO-incompatible KT, and of five (2.5%) patients who had a primary loss of KT, the remaining 198 recipients defined our study cohort. Of these primary non-function cases, four (1.9%) were LRD recipients and one (0.5%) was a LURD recipient (p=0.650).

Baseline data and graft outcomes

Baseline demographic, anthropomorphic, analytical, and clinical data were collected from both recipients and donors. Transplant data were also analyzed. Human leukocyte antigen (HLA) -incompatible KT refers to cases in which transplants are performed in the presence of preformed donor-specific antibodies (DSA). The Chronic Kidney Disease Epidemiology Collaboration (CKD-EPI) equation was used to predict the estimated glomerular filtration rate (eGFR). Delayed graft function (DGF) was defined as the need for dialysis in the first week after transplantation. Graft biopsies were performed for indication. Each recipient was followed up until the end of June 2019, the date of death, graft loss, or loss during follow-up. The study protocol was reviewed and approved by the institutional ethical review and hospital administration boards (approval no. 147/21 [119-DEFI/122-Ce]) as per the recommendations of the Declaration of Helsinki and European Data Protection Regulations.

Immunosuppression (IS) and desensitization protocols

Induction therapy was used in most patients, with an anti-interleukin (IL)-2 receptor monoclonal antibody (basiliximab (Novartis), 20 mg twice on days 0 and 4) or polyclonal anti-thymocyte globulin (ATG) (Fresenius, 3 mg/kg for five to seven days). The ATG was primarily used in HLA-incompatible KT and retransplants. All enrolled recipients had similar triple maintenance immunosuppression consisting of oral tacrolimus, mycophenolate mofetil (MMF), and methylprednisolone (MP)/prednisolone. Further details of our regimen have already been published [16].

The HLA-incompatible KT received desensitization with intravenous immunoglobulin (2 g/kg) at transplant (0.5 g/kg immediately before transplant, and on days 1, 2, and 3) and one month after transplantation (1 g/kg in two consecutive days), and a dose of rituximab (375 mg/ m2) on day 3 post-transplant. Given the strength of preformed anti-HLA DSA and flow cytometry crossmatch results, six patients also underwent plasmapheresis every other day (the first session three days before the transplant, for a total of six to nine sessions) [16].

Statistical analysis

Continuous data are described as mean ± standard deviation (SD) or median (interquartile range (IQR)), and categorical data are expressed as numbers and percentages. Categorical data were compared using Pearson’s chi-square test or Fisher’s exact test, and continuous variables were compared using Student’s t-test or Mann-Whitney U-test, as appropriate.

The AR and graft survival curves were visualized using the Kaplan-Meier method. Comparisons between patient groups were performed using the log-rank test. In cases of death with a functioning graft, the time was censored at the time of death. Potential predictors of AR and graft failure were explored using univariate and multivariate Cox proportional hazard models. In all multivariable models, independent predictors were identified using a backward elimination method, with a P-value < 0.05 necessary for retention in the model.

A two-sided P-value of < 0.05 was considered statistically significant. Statistical calculations were performed using Stata that can perform symmetric multiprocessing (STATA/MP), version 15.1 (Stata Corp, College Station, TX, USA).

## Results

Characteristics of patients

Our study cohort comprised 198 recipients; 59% (n=116) had LRD KT, and 41% (n=82) had LURD KT. The main group characteristics of living donor pairs and transplants based on LRD and LURD are shown in Table 1.

**Table 1:** Baseline characteristics of living donor pairs and transplants, based on LRD and LURD. LRD: Living related donor; LURD: Living unrelated donor; R: Recipient; SD: Standard deviation; F: Female; BMI: Body mass index; KT: Kidney transplant; IQR: Interquartile range; RRT: Renal replacement therapy; HD: Hemodialysis; PD: Peritoneal dialysis; D: Donor; eGFR: Estimated glomerular filtration rate; PRA: Panel-reactive antibodies; HLA: Human leukocyte antigen; MM: Mismatch; ATG: Antithymocyte globulin; TAC: Tacrolimus; MMF: Mofetil mycophenolate; pred: Prednisone; DGF: Delayed graft function

	Total N=198	LRD N=116 (59%)	LURD N=82 (41%)	P-value
Recipient				
Age of R, mean ±SD	41.1±13.2	35.9±12.2	48.5±10.9	<0.001
Sex of R (F), n (%)	56 (28)	36 (31)	20 (24)	0.307
BMI of R, mean±SD	23.9±3.9	23.2±3.9	24.9±3.9	0.004
Time on dialysis before KT (months), median (IQR)	13.9 (0-30.3)	12.6 (0-27.2)	16.3 (3.9-32.3)	0.125
RRT pre-KT, n (%)				0.061
Preemptive	50 (25)	33 (28)	17 (21)	
HD	106 (54)	54 (47)	52 (63)	
PD	42 (21)	29 (25)	13 (16)	
Donor				
Age of D, mean ± SD	48.1±10.5	47.4±11.4	49.1±9.2	0.265
Sex of D (F), n (%)	143 (72)	80 (69)	63 (77)	0.224
BMI of D, mean±SD	25.3±3.5	25.2±3.5	25.4±3.5	0.798
Predonation eGFR, mean ± SD	100.2±14.3	101.4±14.0	98.4±14.6	0.144
Left kidney donated, n (%)/Missing: 9	156 (83)	89 (81)	67 (85)	0.486
Transplant				
Year of KT, n (%)				0.903
2008-2012	71 (36)	42 (36)	29 (35)	
2013-2017	127 (64)	74 (64)	53 (65)	
Retransplant, n (%)	27 (14)	18 (16)	9 (11)	0.359
Calculated PRA >0%, n (%)	60 (30)	37 (32)	23 (28)	0.562
HLA-incompatible KT, n (%)	22 (11)	14 (12)	8 (10)	0.610
HLA-A MM, mean±SD	0.94±0.68	0.63±0.52	1.39±0.62	<0.001
HLA-A MM, n (%)				<0.001
0	51 (26)	45 (39)	6 (7)	
1	107 (54)	69 (59)	38 (46)	
2	40 (20)	2 (2)	38 (46)	
HLA-B MM, mean±SD	1.16±0.72	0.80±0.64	1.66±0.50	<0.001
HLA-B MM, n (%)				<0.001
0	38 (19)	37 (32)	1 (1)	
1	91 (46)	65 (56)	26 (32)	
2	69 (35)	14 (12)	55 (67)	
HLA-DR MM, mean±SD	0.97±0.69	0.67±0.59	1.40±0.61	<0.001
HLA-DR MM, n (%)				<0.001
0	50 (25)	45 (39)	5 (6)	
1	103 (52)	64 (55)	39 (48)	
2	45 (23)	7 (6)	38 (46)	
IS Induction, n (%)				0.614
Without	5 (3)	4 (3)	1 (1)	
Basiliximab	172 (87)	100 (86)	72 (88)	
ATG	21 (11)	12 (10)	9 (11)	
Maintenance IS, n (%)				1
TAC + MMF+pred	192 (97)	112 (97)	80 (98)	
Others	6 (3)	4 (3)	2 (2)	
DGF, n (%)	8 (4)	5 (4)	3 (4)	1
Follow-up (years), median (IQR)	5.1 (3.3-7.2)	5.2 (3.4-8.3)	4.9 (3.0-6.9)	0.327

The mean recipient age at the time of KT was lower in LRD compared to LURD recipients (35.9±12.2 vs. 48.5±10.9 years old, p< 0.001). The percentage of preemptive KT was 28% (n=33) in the LRD group and 21% (n=17) in the LURD group (p=0.061). The HLA-A mismatches (MM) were significantly higher in LURD recipients at 1.40±0.61 compared to 0.67±0.59 in LRD recipients. The HLA-B MM was also higher in LURD recipients at 1.66±0.50 compared to 0.80±0.64 in LRD recipients, as well as HLA-DR MM, which was 0.67±0.59 for LURD recipients and 0.67±0.59 for LRD recipients (P<0.001 for all HLA MM comparisons). 

The immunosuppression induction regimen included basiliximab in 86% (n=100) of LRD recipients and 88% (n=72) of LURD recipients, ATG in 10% (n=12) of LRD recipients and 11% (n=9) of LURD recipients; 3% (n=4) of LRD recipients, and 1% (n=1) of LURD recipients had no induction immunosuppression (p=0.614). The maintenance immunosuppression regimen included triple immunosuppression with tacrolimus, mofetil mycophenolate, and prednisone in 97% (n=113) of the LRD recipients and 98% (n=79) of the LURD recipients. Median follow-up was of 5.0 years (IQR: 3.3-7.2).

Acute rejection

**Figure 1:**
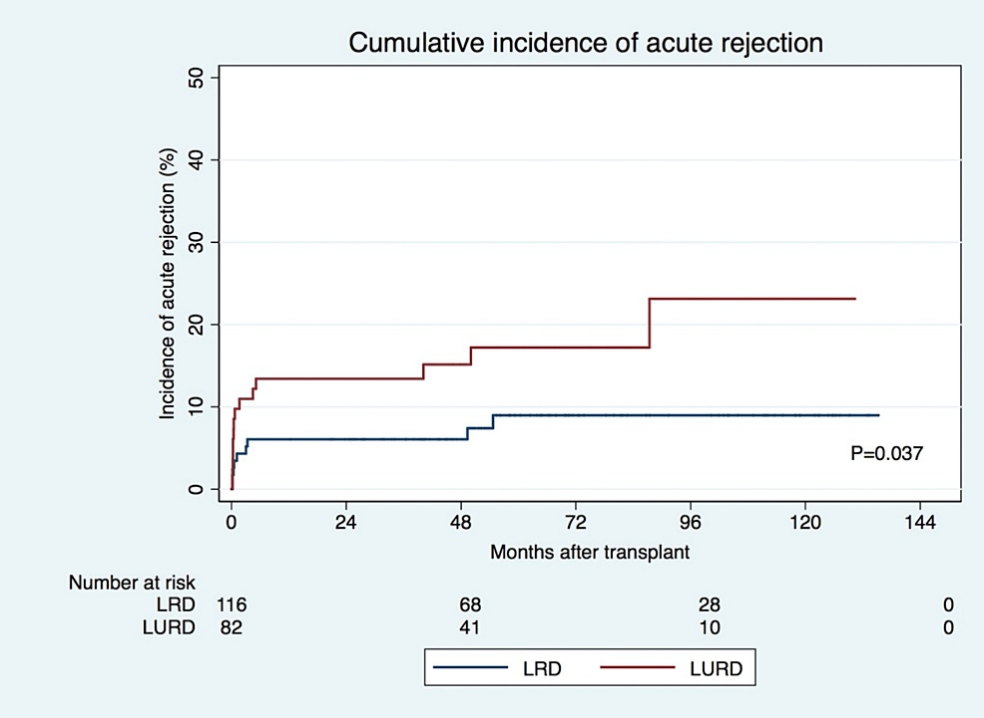
Cumulative incidence of acute rejection according to LRD or LURD KT LRD: Living related donor; LURD: Living unrelated donor; KT: Kidney transplant

The AR was observed in 23 recipients (12%), of which 13 had acute cellular rejection (ACR) and 10 had antibody-mediated rejection (ABMR) (Table 2). Nine cases (8%) of AR were identified in LRD recipients, while LURD recipients had 14 cases (17%) of AR (p=0.044). The cumulative incidence of AR during the follow-up period is shown in Figure 1. The days until AR and the incidence of ABMR and T cell-mediated (cellular) rejection (TCMR) were similar in both groups.

**Table 2:** Impact of LURD vs. LRD transplants in acute rejection LRD: Living related donor; LURD: Living unrelated donor; AR: Acute rejection; ACR: Acute cellular rejection; ABMR: Antibody-mediated rejection; IQR: Interquartile range

	Total N=198	LRD N=116 (59%)	LURD N=82 (41%)	P-value
Acute rejection (AR) n (%)	23 (12)	9 (8)	14 (17)	0.044
Days to AR, median (IQR)	21 (9-154)	34 (12-100)	18 (9-154)	0.900
ACR (%)	13 (7)	5 (4)	8 (10)	0.128
Days to ACR, median (IQR)	16 (12-91)	34 (16-91)	14 (7-92)	0.305
Antibody-mediated rejection (ABMR), n (%)	10 (5)	4 (3)	6 (7)	0.324
Days to ABMR, median (IQR)	61 (9-1480)	53 (6-790)	613 (9-1502)	0.284

In the univariate analysis, LURD recipients were at an increased risk of AR (HR=2.348; p=0.046). However, multivariate analysis showed that LURD was not an independent risk factor for AR (after adjustment for the recipient and donor age, sex, immunosuppression induction regimen, previous time on kidney replacement therapy (KRT), type of previous KRT, re-transplantation rate, donor eGFR, the prevalence of HLA-A MM, HLA-B-MM, and receptor BMI) (Table 3). In contrast, HLA-DR MM increased the HR of AR in both groups (HR=2.256, p=0.011). Both HLA-A and HLA-B MM did not affect the AR HR between the groups of patients. Additionally, KT occurring in the 2008 to 2012 time frame was associated with a significantly higher risk of AR (HR=2.480, p=0.039).

**Table 3:** Predictors of acute rejection *Adjusted to recipient age, donor age, sex, induction of IS therapy, IS maintenance, retransplant rate, months on RRT, type of dialysis/preemptive, donor eGFR, HLA A MM, HLA B MM, KT HLAi, and recipient BMI. HR: Hazard ratio; LURD: Living unrelated donor; HLA: Human leukocyte antigen; HLAi: Human leukocyte antigen incompatibility; MM: Mismatch; KT: Kidney transplant; IS: Immunosuppression; BMI: Body mass index; RRT: Renal replacement therapy; eGFR: Estimated glomerular filtration rate; CI: Confidence interval

	HR (CI 95%)	P-value
Univariate analysis		
LURD	2.348 (1.016-5.427)	0.046
Multivariate analysis 1*		
HLA-DR MM	2.256 (1.205-4.223)	0.011
Year of KT, 2008-2012	2.480 (1.047-5.874)	0.039

When ACR and ABMR were analyzed separately, higher BMI was associated with a higher risk of ACR (HR=1.179, p=0.013) (Table 4), and HLA-DR MM had an independent impact on ABMR (HR=2.892, p=0.045) and HLA incompatibility (HR=5.070, p=0.012) (Table 5). The LURD KT was not significantly associated with any of the rejection types in either univariate or multivariate analysis.

**Table 4:** Predictors of acute cellular rejection (ACR) * Adjusted to recipient age, donor age,  recipient sex, donor sex, induction of IS therapy, IS maintenance, retransplant rate, months on RRT, type of dialysis/preemptive, donor eGFR, HLA-A MM, HLA-B MM, KT HLAi, and KT time period. HR: Hazard ratio; LURD: Living unrelated donor; HLA: Human leukocyte antigen; HLAi: Human leukocyte antigen incompatibility; MM: Mismatch; KT: Kidney transplant; IS: Immunosuppression; BMI: Body mass index; RRT: Renal replacement therapy; eGFR: Estimated glomerular filtration rate; CI: Confidence interval

	HR (CI 95%)	P-value
Univariate analysis		
LURD	2.345 (0.767-7.169)	0.135
Multivariate analysis*		
BMI R	1.179 (1.035-1.344)	0.013

Graft and patient survival

**Figure 2:**
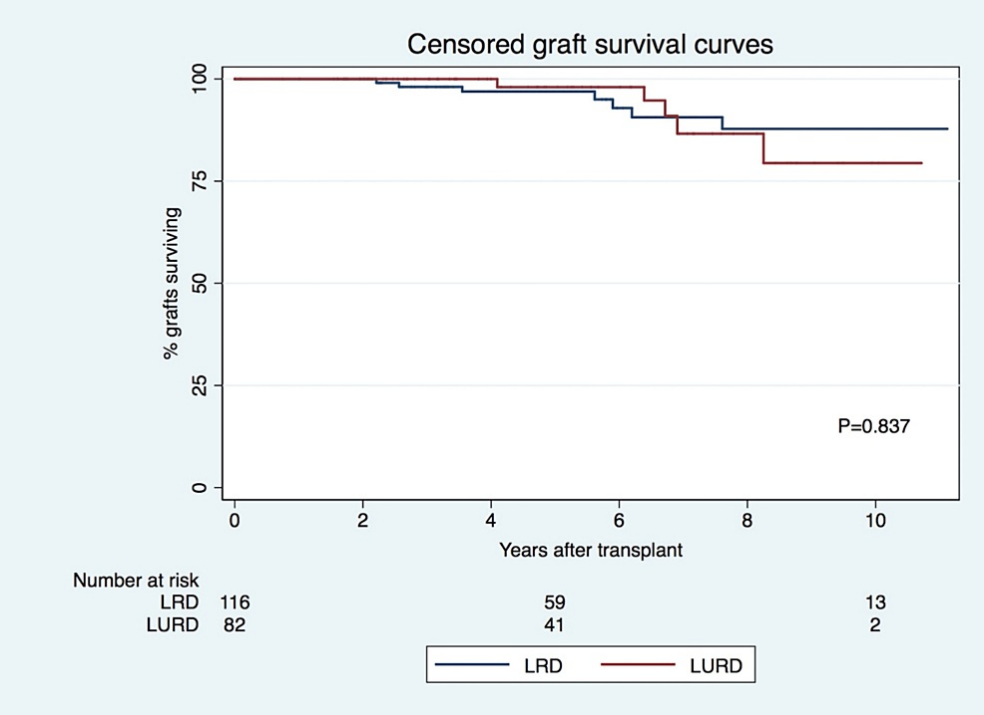
Censored graft survival for LRD and LURD recipients LRD: Living related donor; LURD: Living unrelated donor

Censored graft survival was similar for LRD and LURD recipients (96.9% vs. 98.0% at five years and 87.8% vs. 79.4% at 10 years, p=0.837, respectively) (Figure 2), which remained true after adjustment for several factors. Recipient age (HR=0.938, p<0.05), the occurrence of AR (HR 16.576, p< 0.001), and the presence of preformed DSA (HR=3.387, p<0.05) were identified as predictors of censored graft failure (Table 6). Graft survival rates at five and 10 years were 99%/94% for LRD recipients with no AR, 78%/47% for LURD recipients with AR, 100%/100% for LURD recipients with no AR, and 91%/28% for LURD recipients and AR (overall p<0.001). Patient survival was similar in both groups (one (1%) death in the LRD group and one (1%) death in the LURD group, p=0.422).

Graft function

**Figure 3:**
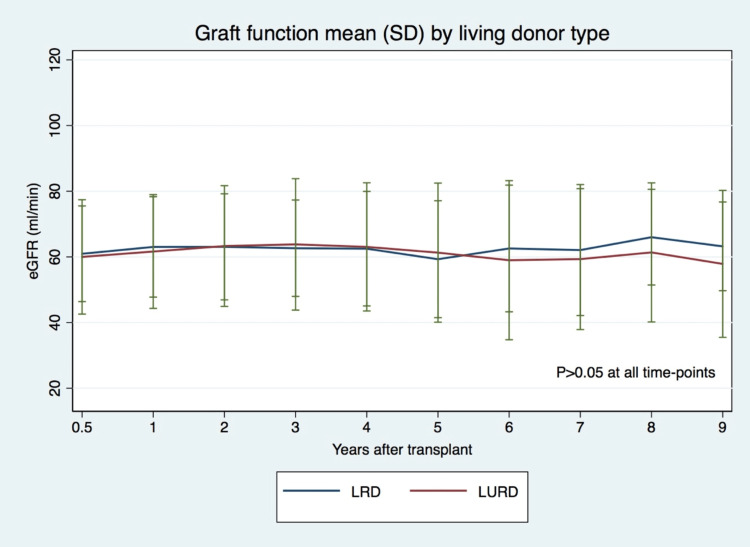
Graft function (eGFR) in LRD and LURD recipients eGFR: Estimated glomerular filtration rate; LRD: Living related donor; LURD: Living unrelated donor; SD: Standard deviation

Graft function was similar in both groups over the follow-up period: around 60-65 mL/min (p>0.05, in all evaluated time-points) (Figure 3).

## Discussion

Living KT can mitigate organ scarcity and help reduce KT waitlists [3]. Evaluating the impact of living donor sources on long-term outcomes may potentially allow the optimization of donor matching and immunosuppression to improve results [17]. In this study, we aimed to compare graft function and survival between LURD and LRD transplant recipients. Additionally, we studied AR occurrence in both groups.

In our study, graft function was similar in both groups during the medium and long-term follow-up periods. Other studies have found that the donor source does not significantly influence graft function in living donation [10,14]. Censored graft survival was also comparable between LURD and LRD patients, which is in line with most previous studies suggesting graft survival to be similar for both groups of patients [7]. In a recent large-scale study, however, LURD had higher death-censored graft failure than LRD recipients [14], which contrasts with our results; although the authors stated that they could not explain this finding, they speculated that it could be due to old age and a high proportion of patients with type II diabetes mellitus and hypertension as primary kidney disease among LURD transplant recipients. Long-term follow-up and large-scale studies are necessary to confirm these results.

Patient survival was also similar between both groups, with one (1%) death in the LRD group and one (1%) death in the LURD group. A United States study from 1998 [12] showed that 10-year patient survival among recipients of LURD transplants was worse than that of LRD transplants (86% vs. 63%, respectively), although these findings have not been replicated in other more recent studies, which have consistently shown similar survival rates between LRD and LURD KT recipients [10,14].

In our study, the univariate analysis showed that LURD recipients had a higher incidence of AR. However, in the multivariate analysis, LURD recipient status was not found to be an independent risk factor for AR, consistent with findings from previous studies [8]. However, the presence of HLA-DR MM predicted an increased risk of AR, regardless of the donor origin. The LURD recipients had higher HLA-DR MM, which explains why, despite having a higher rate of AR in multivariate analysis, LURD recipient status did not predict AR, while HLA-DR MM did (Tables 1, 3). In contrast, HLA-A and HLA-B MM did not affect the AR between the groups. The HLA-MM is recognized as a strong risk factor for the development of AR [18,19] and HLA-DR MM, in particular, has been shown to strongly influence KT outcomes [20], namely AR [21,22]. More recently, HLA-DR epitope mismatch is an independent predictor of ABMR [23]. A recent study using random forest analysis in the United Network for Organ Sharing (UNOS) database identified HLA-DR as an important variable for acute rejection among Black kidney transplant recipients in the United States [24]. In pancreatic transplantation, HLA-DR MM has been shown to independently predict acute rejection [25], an effect that might be reproducible in KT. The number of days until AR after KT was similar between LURD and LRD recipients. Other adverse outcomes are associated with HLA-MM. In the case of deceased donors, HLA matching has been shown to correlate with renal allograft and patient survival, even in the absence of preformed DSA [26,27], but few studies have evaluated this relationship in living donor transplants. In one study with first adult transplants from deceased donors in the United States between 1987 and 2013, a significant linear relationship between HLA MM and graft survival were identified, with one MM conferring a 13% higher risk and six MM conferring a 64% higher risk of allograft failure [26]. In another study, 83 0-HLA MM patients were matched to 407 controls with more than 0-HLA MM, with the authors reporting no differences in death-censored graft survival or patient survival for both groups [28]. Our data reinforce the importance of HLA-DR matching and its association with graft survival and incidence of rejection [25].

The hurdle associated with high HLA MM in LDKT may potentially be managed by the introduction of compatible pairs in a KPE program, which has been possible in Portugal since legislation concerning the National KPE program was amended in 2021. Careful immunological risk profiling, including improved HLA and epitope analysis, could also improve these results. In addition, a clear definition of the inclusion criteria for compatible pairs in KPE is crucial. A recent report that reviewed the first nine years of KPE transplants from the National Kidney Registry in the United States showed a 27% lower five-year graft failure rate compared to traditional direct living donor transplants [29], and improved transplant outcomes have been attributed to improved antibody avoidance. In the setting of an increasingly hypersensitized population of KT candidates worldwide, surely the optimal choice type of KT is a low HLA mismatch transplant. We should also refer to our finding that KT occurring in the 2008 to 2012 time frame was associated with a significantly higher risk of AR (HR=2.480, p=0.039). We hypothesize this is most likely due to changes in our center's immunosuppression protocol, which was mainly based on cyclosporine for the majority of patients before 2012, and increasingly became tacrolimus-based in the most recent years, justifying a higher incidence of AR in the aforementioned period.

In the multivariate analysis with separate ACR and ABMR, BMI was associated with a higher risk of ACR (HR=1.179, p=0.013). Other studies have reported recipient obesity as a risk factor for AR [30]. This has been attributed to more difficult dosage-adjustment immunosuppressant medications in this population and to the low-grade inflammatory state that characterizes obesity, which has also been hypothesized as a factor that may impact the efficacy of immunosuppressants. Adapted immunosuppressant targets/regimens to the obese population might improve results in this specific group of patients, in the future.

In our population, the preemptive KT rate was similar in both groups, 28% (n=33) for LRD recipients and 21% (n=12) for LURD recipients. As waiting time on dialysis is considered the strongest modifiable risk factor for KT outcomes, increasing this rate would certainly improve our results.

The major limitation of this study is the sample size. However, it should be emphasized that our data refers to a single-center population, with similar background demographic and clinical features, submitted to KT by the same multidisciplinary team, which delivered the same standards of patient care. This allowed the retrieval of robust data for statistical analysis; thus, valid conclusions can still be ascertained. In addition, the fact that this was a retrospective study rather than a prospective study designed to assess KT outcomes with formal event adjudication implies that the level of evidence is not as high as would be derived from a clinical trial. On the other hand, our results are significant, as our center is currently responsible for more than half of all LDKT performed in Portugal.

## Conclusions

In conclusion, our study of LURD and LRD KT recipients showed similar graft function over time and similar censored-death survival rates. The AR was higher in LURD recipients compared to LRD recipients. However, multivariate analysis showed that LURD recipient status was not an independent risk factor for AR. The HLA-DR MM was an independent predictor of AR, while HLA-A and HLA-B MM did not affect AR HR between groups of patients, reinforcing the importance of HLA-DR matching and its association with graft survival and incidence of rejection.
